# Polystyrene nanoplastics with different functional groups and charges have different impacts on type 2 diabetes

**DOI:** 10.1186/s12989-024-00582-w

**Published:** 2024-04-24

**Authors:** Yunyi Wang, Ke Xu, Xiao Gao, Zhaolan Wei, Qi Han, Shuxin Wang, Wanting Du, Mingqing Chen

**Affiliations:** Hubei Key Laboratory of Genetic Regulation and Integrative Biology, School of Life Sciences, Central China Normal University, 430079 Wuhan, Hubei China

**Keywords:** AKT, FoxO1, Functional groups, Nanoplastics, Type 2 diabetes

## Abstract

**Background:**

Increasing attention is being paid to the environmental and health impacts of nanoplastics (NPs) pollution. Exposure to nanoplastics (NPs) with different charges and functional groups may have different adverse effects after ingestion by organisms, yet the potential ramifications on mammalian blood glucose levels, and the risk of diabetes remain unexplored.

**Results:**

Mice were exposed to PS-NPs/COOH/NH_2_ at a dose of 5 mg/kg/day for nine weeks, either alone or in a T2DM model. The findings demonstrated that exposure to PS-NPs modified by different functional groups caused a notable rise in fasting blood glucose (FBG) levels, glucose intolerance, and insulin resistance in a mouse model of T2DM. Exposure to PS-NPs-NH_2_ alone can also lead the above effects to a certain degree. PS-NPs exposure could induce glycogen accumulation and hepatocellular edema, as well as injury to the pancreas. Comparing the effect of different functional groups or charges on T2DM, the PS-NPs-NH_2_ group exhibited the most significant FBG elevation, glycogen accumulation, and insulin resistance. The phosphorylation of AKT and FoxO1 was found to be inhibited by PS-NPs exposure. Treatment with SC79, the selective AKT activator was shown to effectively rescue this process and attenuate T2DM like lesions.

**Conclusions:**

Exposure to PS-NPs with different functional groups (charges) induced T2DM-like lesions. Amino-modified PS-NPs cause more serious T2DM-like lesions than pristine PS-NPs or carboxyl functionalized PS-NPs. The underlying mechanisms involved the inhibition of P-AKT/P-FoxO1. This study highlights the potential risk of NPs pollution on T2DM, and provides a new perspective for evaluating the impact of plastics aging.

**Supplementary Information:**

The online version contains supplementary material available at 10.1186/s12989-024-00582-w.

## Background

A major environmental issue on a global scale is plastic pollution. It is estimated that 11 billion metric tons of plastic waste will pollute the natural environment by 2025 [1]. In the environment, plastics can be broken down into microplastics (MPs), and even smaller nanoplastics (NPs) through weathering, biodegradation and photodegradation [2]. People are exposed to plastic particle pollution through inhalation, ingestion and dermal contact [3]. Compared to MPs, NPs with higher specific surface area. are more likely to enter cells and tissues [4], and therefore have greater potential to threaten physical health. NPs have demonstrated the ability to cross significant physiological barriers, thereby presenting additional potential risks to human health [5]. *In vitro* studies have identified a variety of toxic effects caused by Micro/Nanoplastics (MNPs) exposure, on mammalian cells including cytotoxicity, oxidative stress, endoplasmic reticulum stress, apoptosis, inflammatory response and genotoxicity [6]. Bioaccumulation of MNPs has also been confirmed in *in vivo* rodent models in the liver, intestines, kidney, brain, spleen, and lung, resulting in negative effects of varying severity, including reproductive toxicity, neurotoxicity, and metabolic toxicity [7].

Mounting evidence underscores the toxicity of NPs, however, most of these studies have concentrated on unaltered, single plastic particles. It can be quite difficult to determine how dangerous plastic particles can be to organisms, since the harm can be influenced by a number of factors, such as the biological subjects studied, the exposure concentration, and the exposure conditions, as well as the form, size, and type of plastic [8–10]. Plastic particles that are the result of aging in the environment can carry different functional groups. The carboxyl (-COOH) and amino (-NH_2_) on the surface of polystyrene nanoparticles (PS-NPs) are common modified functional groups, with negative and positive charges, respectively [11]. These charged particles can pass through cell membranes more easily, because they resemble proteins in terms of their chemical structure [12]. Qu et al. proved that amino-modification increased the harmful effects of nano-polystyrene on gonad growth and reproductive ability in *C. elegans* [13]. Liu et al.‘s exposure experiments on human cervical cancer cells using 50 nm PS-NPs, revealed that amino-functionalized and carboxyl-functionalized PS-NPs could alter the cell cycle more significantly than non-functionalized PS-NPs [14]. Functionalized PS-NPs were more likely to aggregate, and exacerbated apoptosis compared to pristine PS [15]. In *Arabidopsis thaliana*, NPs with positive charges, and NPs with negative charges were able to accumulate, but the uptake of positively charged NPs was somewhat limited compared with negatively charged NPs [16]. In addition, marine clams (*Meretrix meretrix*) were shown to have an imbalance in their energy homeostasis and an unbalanced immune system because of the bioaccumulation of charged PS-NPs [17]. Teng et al. showed that the adverse effects caused by PS-NPs on the development and behavior of zebrafish are specifically altered by the effects of surface charge [18]. The above-mentioned studies suggest that different charges and functional groups may enhance NPs bioconcentration, and may have different adverse effects on organisms.

Type 2 diabetes mellitus (T2DM), which accounts for 90% of all diabetes casas, is a common metabolic condition and one of the most serious global health threats [19]. It is a multi-cause chronic disease characterized by insulin resistance (IR). IR is characterized by a low efficiency of insulin secretion and utilization, thus inducing sustained higher blood glucose levels [20]. Insulin can regulate carbohydrates and lipids through the PI3K/AKT signaling pathways. In pancreatic β-cells, AKT is essential for survival, proliferation and normal physiological functions [21–23]. Mao et al. showed that the AKT/FOXO1 signaling pathway is closely related to genes involved in gluconeogenic adipogenesis, and that the phosphorylation level of AKT/FOXO1 was reduced in the diabetes model group [24].

Existing studies have indicated that MNPs can cause abnormal glucose and lipid metabolism [1, 25]. Shi et al. indicated that exposure to PS-MPs induced an increase in IR in mice [26]. In our recent research, we showed that PS-NPs exposure aggravated IR and glucose intolerance in T2DM model mice [27]. However, the relationship between NPs pollution and the risk of diabetes remains unclear, and epidemiological studies and animal toxicology research into this relationship is lacking. A literature search revealed that studies examining the impact of NPs with various functional groups and charges on blood glucose levels, have not produced any important findings. For this study we therefore aim at comparing the impacts of exposure to PS-NPs with different functional groups and charges on blood glucose levels and IR in mice. We also explored the mechanism underlying their effects on T2DM through activating the AKT/FOXO1 signaling pathway using SC79, a selective AKT activator.

## Methods

### Animals

One hundred and twenty male C57BL/6 mice were ordered from the Hubei Provincial Laboratory Animal Center. The mice, 4–5 weeks old, were housed in pathogen-free conditions at 25 ± 1℃ with 50 ± 10% air moisture and a 12 h–12 h light-dark cycle. Standard diet and germfree water were freely available. All relevant experiments were carried out in accordance with ethical approval (CCNU-IACUC-2022-005).

### Reagents and antibodies

The 80 nm functionalized PS-NPs: PS-NP-COOH; PS-NP; and PS-NP-NH_2_ were obtained from Tianjin Saierqun Technology Co., Ltd (Tianjin, China). Streptozocin (STZ) was obtained from Solarbio Science & Technology Co., Ltd (Beijing, China). The ELISA kits for insulin were obtained from Enzyme-linked Biotechnology Co., Ltd (Shanghai, China). The Glutathione (GSH) detection kits (KTB1600) were purchased from Abbkine. Vazyme Biotech (Nanjing, China) provided the BCA kits. The polyclonal antibodies, P-AKTser473 and P-FoxO1ser256 were obtained from Proteintech and Signalway Antibody, respectively. SC79 used to activate the AKT pathway was purchased from MedChem Express (Shanghai, China). Insulin (Cattle) came from Abmole-China.

### Experimental design

Firstly, the monodisperse polystyrene particles were dispersed in normal saline. and then subjected to ultrasonic vibration for 1 h. The 120 C57BL/6 mice were randomly divided into 12 groups (*n* = 10): (1) saline control (Saline); (2) 5 mg/kg/day PS-NP-COOH (NP-COOH); (3) 5 mg/kg/day PS-NPs (NPs); (4) 5 mg/kg/day PS-NP-NH_2_ (NP-NH_2_); (5) saline + high fat diet (Fat-STZ-Saline); (6) 5 mg/kg/day PS-NP-COOH + high fat diet (Fat-STZ-NP-COOH); (7) 5 mg/kg/day PS-NPs + high fat diet (Fat-STZ-NPs); (8) 5 mg/kg/day PS-NP-NH_2_ + high fat diet (Fat-STZ-NPs-NH_2_); (9) saline + high fat diet + SC79 (Fat-STZ-Saline-SC79); (10) 5 mg/kg/day PS-NP-COOH + high fat diet + SC79 (Fat-STZ-NP-COOH-SC79); (11) 5 mg/kg/day PS-NPs + high fat diet + SC79 (Fat-STZ-NPs-SC79); (12) 5 mg/kg/day PS-NP-NH_2_ + high fat diet + SC79 (Fat-STZ-NPs-NH_2_-SC79). The detailed design of the experiment is shown in Fig. S1. After one week of adaptation, the mice in the exposure groups were orally exposed to 5 mg/kg/day of PS-NPs for 9 weeks, and the control group was given a corresponding dose of saline. The intraperitoneal injection of streptozotocin (STZ) on the basis of feeding high fat diet is a common and mature method to induce type 2 diabetes. Here, the T2DM was built by intraperitoneal injection of small doses of STZ (40 mg/kg/day) every two days and a high-fat diet as described as our previous study [27]. The mice with hyperglycemia were regarded as successful models. In the activator groups, the mice were given 10 mg/kg/d SC79 intraperitoneal injection every three days. The mice were terminally anesthetized with sodium pentobarbital, following which the heart blood and organs were collected. Keep the blood at 20–25℃ for a few hours, and subsequently centrifuged (3000 rpm, 25℃) to collect the serum.

### Determining blood glucose and insulin levels

Glucose concentration in tail vein blood was determined by using a glucose dehydrogenase method as described in previous research [27]. In brief, from week one to week nine, we collected blood taken from the tail vein to measure the FBG levels once a week, using a glucometer (Roche). Mice fasted for 12 h in advance. ELISA kits were used to determine insulin levels in the serum.

### Oral glucose tolerance test (OGTT)

As for a previous study, 2 h-OGTT was administered for each group after the last exposure [28]. The mice fasted for about 12 h, but had free access to water. They were then given an oral dose of glucose solution (2 g/kg), following which blood glucose levels were determined using glucometer at 0, 30, 60, and 120 min intervals. Using the collected data, the blood glucose change curves for the deferent groups were drawn. The area under the curve (AUC) was calculated to indicate glucose tolerance.

### Insulin tolerance test (ITT)

This procedure was similar to that used for determining OGTT. The FBG levels were determined, following which the mice were injected with insulin. After 30, 60 and 120 min, blood glucose levels were again measured, using a glucometer. The glucose change curves were similarly plotted as line graphs. The larger the ITT AUC, the more severe was the insulin resistance of the mice.

### HOMA-IR

The data for FBG, and the fasting insulin quantitative analysis obtained above were used to calculate an insulin resistance index [29]. The equation used to calculate insulin resistance indexes was: HOMA-IR = (C_FINS_ × C_FBG_) ÷ 22.5, where C_FINS_ is the fasting serum insulin level (mIU/L), C_FBG_ is the FBG level (mmol/L).

### Testing for ROS, MDA and GSH

Liver tissue was pre-homogenized in PBS buffer, and centrifuged at 4℃, 15,000 rpm for 15 min. The supernatant was collected and stored at -80℃ to determine biomarkers of oxidative stress in the liver tissue. The levels of ROS and MDA were determined using the DCFH-DA method and the thiobarbituric acid reactive method, respectively, which are described in our previous study [27]. The GSH levels in the liver tissue were measured using GSH kits. A BCA kit was used to determine protein levels in the liver tissue.

### Organ coefficient and pathological histology analysis

The collected organs were placed in PBS buffer to wash away the blood after euthanasia. Filter paper was used to absorb any moisture on the surface. Then the fresh organs were weighed separately, to compute the percentage of organ weight/body weight. The liver and pancreas from three different mice per group were embedded in paraffin after the tissues were washed in PBS and kept in 4% paraformaldehyde for more than 24 h at 25 °C. The paraffin-embedded tissues were cut into 4 μm thick slices. Hematoxylin and Eosin (H&E) counterstaining was then applied on these tissue sections for examination under a microscope (Leica DM 4000B, Germany). Periodic Acid-Schiff (PAS) staining was done following a similar procedure.

### Immunohistochemistry of P-AKTser473 and P-FoxO1ser256

Three sections of mouse liver tissue from each group were routinely processed and then incubated with the phosphorylation antibodies: P-AKTser473 and P-FoxO1ser256 at 4℃ for 12 h, and then reacted with an appropriate HRP-antibody. The reaction product was colored with diaminobenzidine (DAB) complex. The sections were rinsed, counter-stained with hematoxylin, and dried before observation.

### Statistical analyses

One-way analysis of variance was used to analyze the differences between groups. Tukey’s test and the Student’s t-test were applied on multiple comparisons. It was regarded as significant when *p* < 0.05 and *p* < 0.01 was regarded as very significant. GraphPad Prism 8.0 (San Diego, CA, USA) was used to draw statistical graphs. The IOD of each section was calculated using Image Pro Plus 6.0.

## Results

### Effects of PS-NPs modified by different functional groups on body weight and liver index

**Fig. 1:**
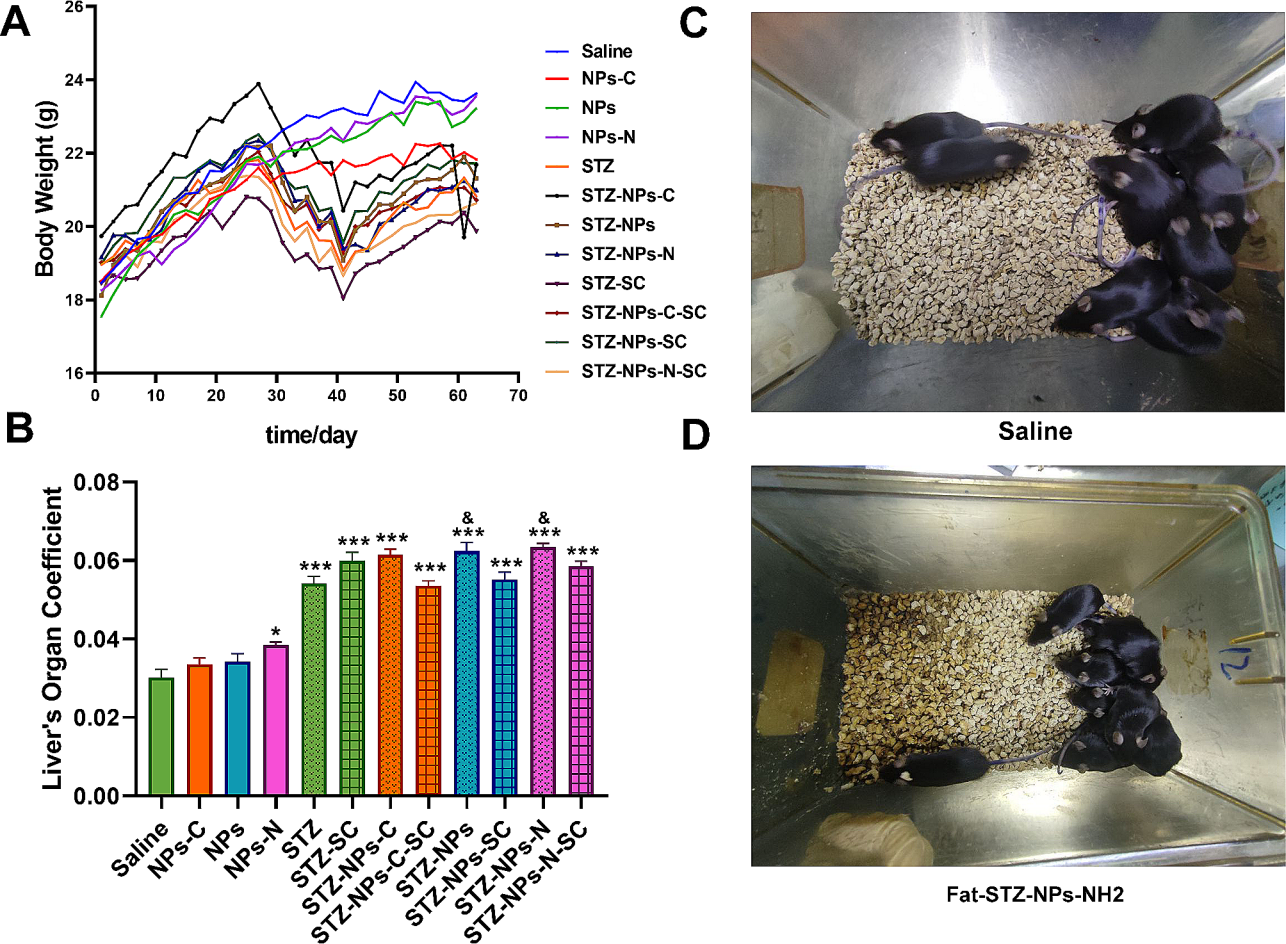
Effects of PS-NPs with different charges on body weight and liver organ coefficient. (**A**) Body weight; (**B**) Organ coefficient of the liver; (**C**) Mice in saline group; (**D**) Mice in Fat-STZ-NPs-NH_2_ group. All pictures were taken on the last day before the mice were euthanized. In (**A**) and (**B**), Saline, NPs-C, NPs, NPs-N, STZ, STZ -SC, STZ - NPs-C, STZ - NPs-C-SC, STZ - NPs, STZ - NPs -SC, STZ - NPs-N, and STZ-NPs-N-SC represent Saline group, NPs-COOH group, NPs group, NPs-NH_2_ group, Fat-STZ-Saline group, Fat-STZ-Saline-SC79 group, Fat-STZ-NP-COOH group, Fat-STZ-NP-COOH-SC79 group, Fat-STZ-NPs group, Fat-STZ-NPs-SC79 group, Fat-STZ-NPs-NH_2_ group and Fat-STZ-NPs-NH_2_-SC79 group, respectively. Results are presented as the mean ± SEM, *n* = 10. The significance of differences was determined by using a one-way ANOVA, combined with multiple comparison tests between Tukey’s groups and Student’s t-test. * means *p* < 0.05, ** means *p* < 0.01, *** means *p* < 0.001 compared with the saline-vehicle control group; & means *p* < 0.05, && *p* < 0.01 compared with Fat-STZ diabetic group; # means *p* < 0.05, ## means *p* < 0.01, ### means *p* < 0.001 compared with the Fat-STZ-NPs/NH_2_/COOH-SC79; $ means *p* < 0.05, $$ means *p* < 0.01, $$$ means *p* < 0.001 compared between exposure groups; ns means no significant difference

The characteristics of the NPs are shown in (Fig. S2). The microstructure and morphology of PS-NP-COOH, PS-NP, and PS-NP-NH_2_ were observed by scanning electron microscope (Fig. S2, A). These three kinds of PS-NPs were confirmed using Fourier-transform infrared spectroscopy (Fig. S2, B) and Zeta potentials analysis (Fig. S2, C). The T2DM mice had lower body weight than those in the non-diabetes model groups on the last day of the exposure (Fig. 1A). In the non-diabetic group, exposure to PS-NPs modified by different charges (functional groups) had no significant effect on their body weight. In the diabetic group, exposure to PS-NPs modified by different charges (functional groups) plus Fat-STZ, made no significant difference to body weight among these T2DM mice. SC79 didn’t effectively alleviate any loss of body weight.

The liver indexes of the diabetes model groups were increased in comparison with those in the non-diabetes model groups (Fig. 1B). In the non-diabetic group, exposure to NPs-NH_2_ induced a significant increase in liver indexes in comparison with the control group (*p* < 0.05). Exposure to NPs/NPs-NH_2_ with Fat-STZ induced a significant increase in comparison with the Fat-STZ group (*p* < 0.05). Whether in T2DM groups or in non-T2DM groups, different charges cause a similar increase in liver indexes. SC79 slightly alleviated the increase in the liver index, as can be seen when comparing Fat-STZ-COOH-NPs, Fat-STZ-NPs, and Fat-STZ-NPs-NH_2_ groups.

### The effect of exposure to PS-NPs modified by different functional groups plus SC79 on fasting blood glucose (FBG) levels

**Fig. 2:**
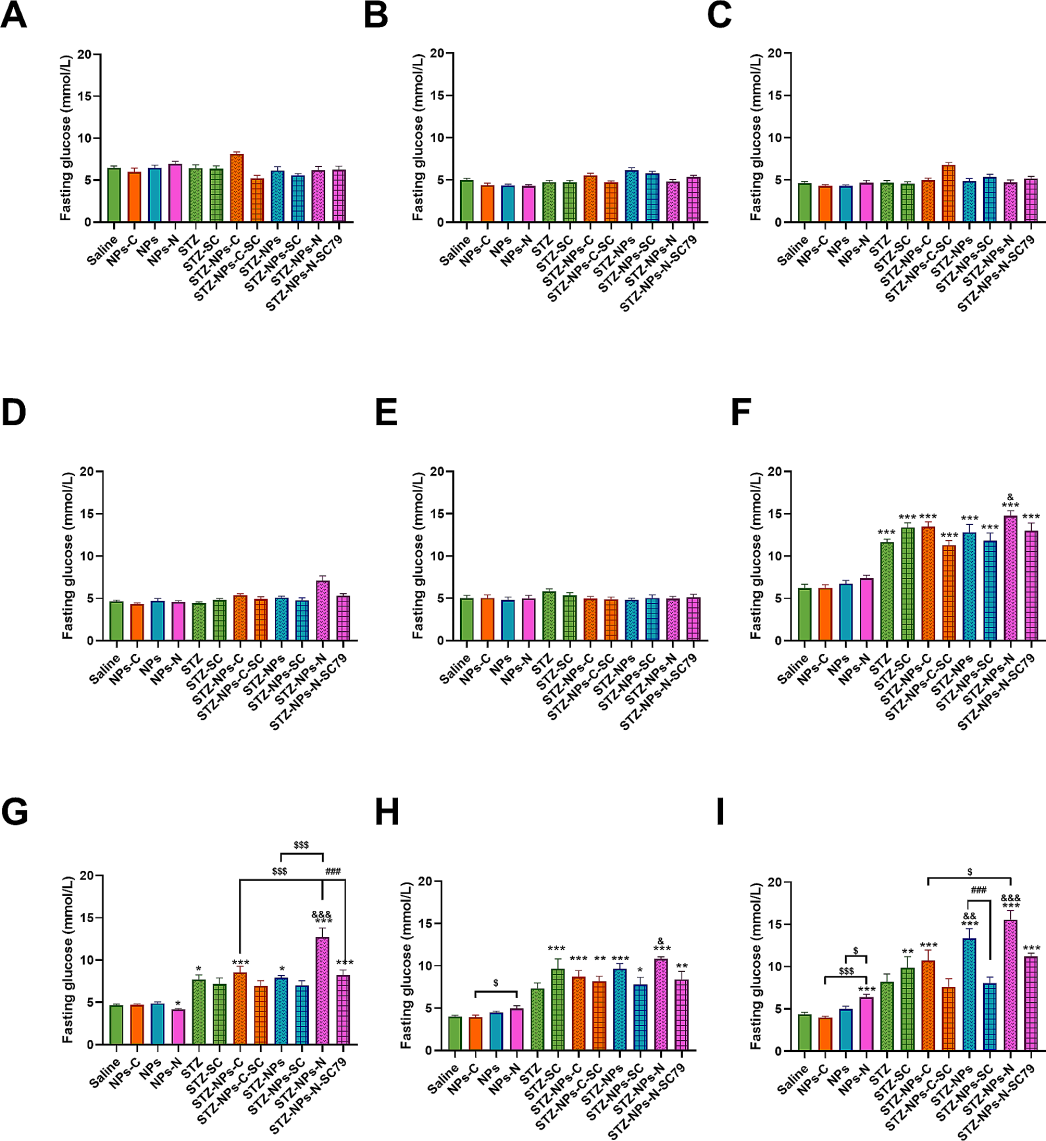
The effect of exposure to PS-NPs with different charges, and SC79 treatment on fasting blood glucose levels. (**A**-**I**) Fasting blood glucose levels. Saline, NPs-C, NPs, NPs-N, STZ, STZ -SC, STZ - NPs-C, STZ - NPs-C-SC, STZ - NPs, STZ - NPs -SC, STZ - NPs-N, and STZ-NPs-N-SC represent Saline group, NPs-COOH group, NPs group, NPs-NH_2_ group, Fat-STZ-Saline group, Fat-STZ-Saline-SC79 group, Fat-STZ-NP-COOH group, Fat-STZ-NP-COOH-SC79 group, Fat-STZ-NPs group, Fat-STZ-NPs-SC79 group, Fat-STZ-NPs-NH_2_ group and Fat-STZ-NPs-NH_2_-SC79 group, respectively. Blood glucose measurements from week 1 to week 9 of the experimental period. Results are presented as the mean ± SEM, *n* = 5–10. Statistical significance was analyzed by One-Way ANOVA among multiple groups. * means *p* < 0.05, ** means *p* < 0.01, *** means *p* < 0.001 compared with the saline-vehicle control group; & means *p* < 0.05, && *p* < 0.01 compared with Fat-STZ diabetic group; # means *p* < 0.05, ## means *p* < 0.01, ### means *p* < 0.001 compared with the Fat-STZ-NPs/NH_2_/COOH-SC79; $ means *p* < 0.05, $$ means *p* < 0.01, $$$ means *p* < 0.001 compared between exposure groups; ns means no significant difference

Figure 2 shows that from the 5th week, the model group showed symptoms of hyperglycemia after the STZ injection, and a significant increase in FBG levels was observed after the 6th week. In the last week, the FBG levels of the NPs-NH_2_ group were remarkably increased in comparison with those of the control group (*p* < 0.001), the NPs-COOH group (*p* < 0.05) and the NPs group (*p* < 0.001). In the diabetic groups, the FBG levels were significantly higher when the T2DM mice were exposed to NPs/NPs-NH_2_ (*p* < 0.01); and the influence of NPs-NH_2_ was the most serious of the three kinds of nanoplastics. Interestingly, after injection with SC79, a specific P-AKTser473 activator, the increase of FBG levels was alleviated when comparing Fat-STZ-NPs with Fat-STZ-NPs- SC79, and Fat-STZ-NPs-NH_2_ with Fat-STZ-NPs-NH_2_-SC79.

### The effect of exposure to PS-NPs modified by different functional groups on OGTT, ITT and insulin resistance

**Fig. 3:**
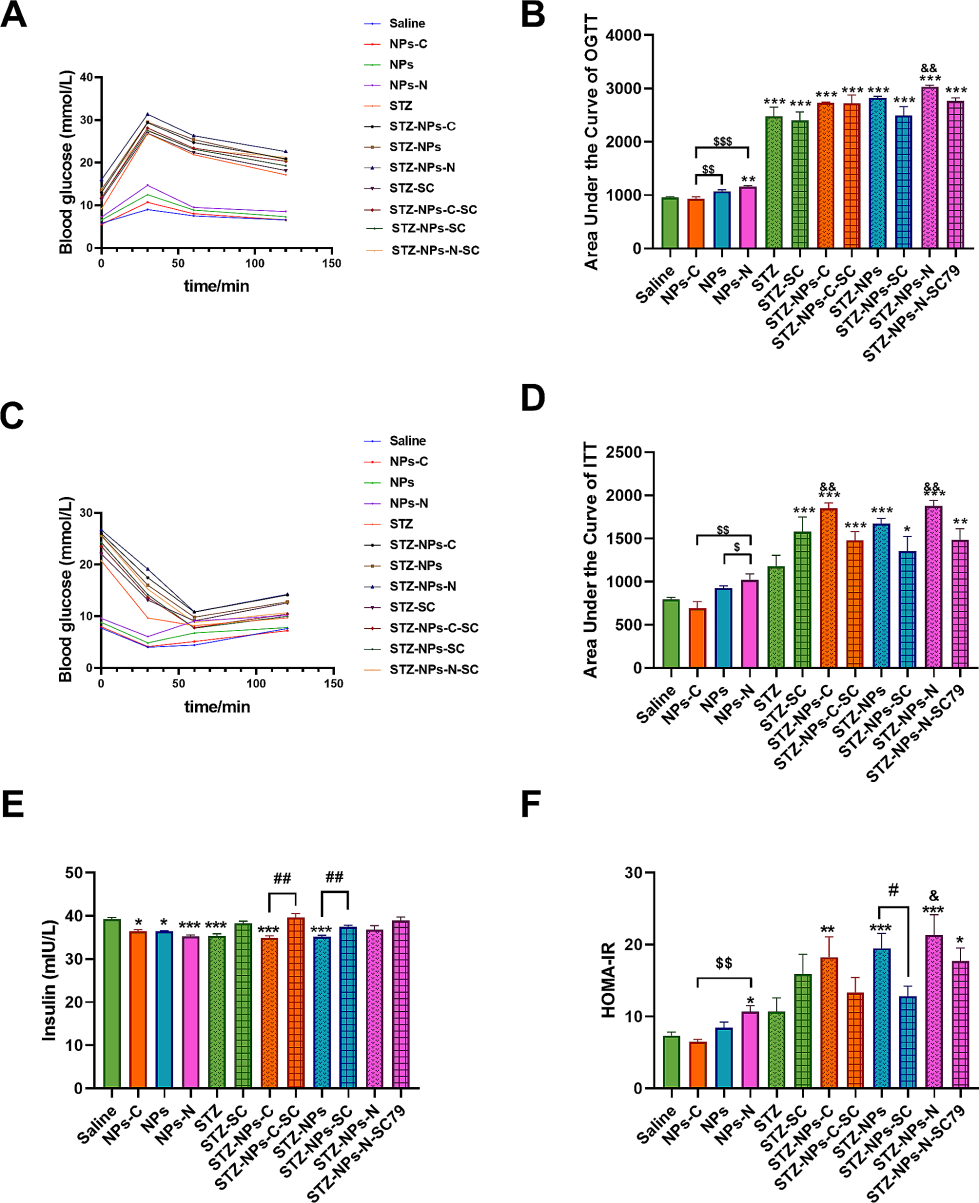
The effect of exposure to PS-NPs with different charges on glucose tolerance (OGTT), insulin tolerance (ITT) and insulin resistance. (**A**) Differences in blood glucose levels over time, after the administration of 2 g/kg glucose; (**B**) Blood glucose area of OGTT under the curve (AUC); (**C**) Differences in blood glucose levels over time after the administration of 0.75 U/kg insulin injection; (**D**) Blood glucose area of ITT under the curve (AUC); (**E**) Serum insulin level; (**F**) HOMA-IR level. Saline, NPs-C, NPs, NPs-N, STZ, STZ -SC, STZ - NPs-C, STZ - NPs-C-SC, STZ - NPs, STZ - NPs -SC, STZ - NPs-N, and STZ-NPs-N-SC represent Saline group, NPs-COOH group, NPs group, NPs-NH_2_ group, Fat-STZ-Saline group, Fat-STZ-Saline-SC79 group, Fat-STZ-NP-COOH group, Fat-STZ-NP-COOH-SC79 group, Fat-STZ-NPs group, Fat-STZ-NPs-SC79 group, Fat-STZ-NPs-NH_2_ group and Fat-STZ-NPs-NH_2_-SC79 group, respectively. Results are presented as the mean ± SEM, *n* = 5–10. AUC was calculated by Area Under the Curve. Statistical significance was analyzed by One-Way ANOVA among multiple groups. * means *p* < 0.05, ** means *p* < 0.01, *** means *p* < 0.001 compared with the saline-vehicle control group; & means *p* < 0.05, && *p* < 0.01 compared with Fat-STZ diabetic group; # means *p* < 0.05, ## means *p* < 0.01, ### means *p* < 0.001 compared with the Fat-STZ-NPs/NH_2_/COOH-SC79; $ means *p* < 0.05, $$ means *p* < 0.01, $$$ means *p* < 0.001 compared between exposure groups; ns means no significant difference

Figure 3A and B show that the AUC of the OGTT of the T2DM groups was higher than that of the non-T2DM groups. In the non-T2DM groups, NPs-NH_2_ treatment induced a remarkable increase in comparison with the control group (*p* < 0.05) and the NPs-COOH/NPs group (*p* < 0.01). In the T2DM groups, only exposure to NPs-NH_2_ induced a significant increase in diabetic mice (*p* < 0.01); SC79 only slightly lessened the increase in the AUC of OGTT, but this was not significant.

In Fig. 3C and D, the AUC of the ITT of the T2DM groups was higher than that of the non-T2DM groups. In the non-T2DM groups, exposure to NPs-NH_2_ induced a minor increase in comparison with the control group, and the ITT index in the NPs-NH_2_ group was clearly higher than the NPs-COOH/NPs group. In the diabetic groups, treatment with NPs-NH_2_ or NPs-COOH could induce a rise in the AUC of the ITT in comparison with the Fat-STZ group (*p* < 0.01). There was no statistical difference among these exposure T2DM groups with different charges (functional groups). After activation of AKT, the increase in the AUC of ITT was attenuated.

Figure 3E shows the insulin levels in the serum. In the non-diabetic groups, treatment with NPs modified by different functional groups induced significant changes when compared with the saline group (*p* < 0.01). In the diabetic group, exposure to NPs/ NPs-COOH/NPs-NH_2_ combined with Fat-STZ resulted in no significant differences in insulin levels in comparison with the Fat-STZ group. Whether in the diabetic groups or non-diabetic groups, different charges caused a similar increase in insulin levels. Treatment with SC79 effectively attenuated the insulin levels that had decreased in the Fat-STZ-NPs-COOH, Fat-STZ-NPs and Fat-STZ-NPs groups (*p* < 0.01).

The diabetes model groups had higher HOMA-IR levels than the non-diabetes model groups (Fig. 3F). In the non-T2DM groups, treatment with NPs-NH_2_ induced a clear increase in HOMA-IR levels when compared with the control group (*p* < 0.05); and the HOMA-IR levels in the NPs-NH_2_ group were clearly higher in comparison with the NPs-COOH group (*p* < 0.01). In the T2DM groups, treatment with NPs-NH_2_ induced a remarkable increase in comparison with the Fat-STZ group (*p* < 0.05); but there were no significant differences among these exposure groups with different charges (functional groups). Treatment with SC79 effectively decreased HOMA-IR levels, which can be seen by comparing Fat-STZ-NPs-COOH-SC79 with Fat-STZ-COOH-NPs, Fat-STZ-NPs- SC79 with Fat-STZ-NPs, and Fat-STZ-NPs-NH2-SC79 with Fat-STZ-NPs-NH2 (*p* < 0.05).

### The impact of exposure to PS-NPs modified by different functional groups on oxidative stress

**Fig. 4:**
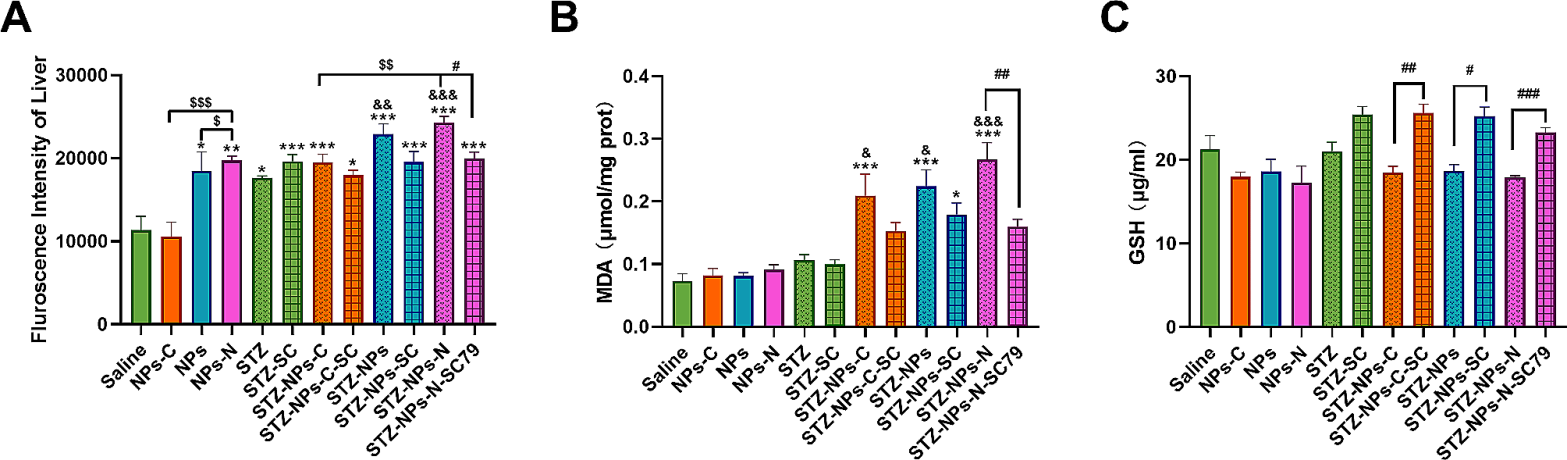
The impact of exposure to PS-NPs with different charges on oxidative stress. (**A**) ROS levels in the liver tissue; (**B**) MDA levels in the liver tissue; (**C**) GSH levels in the liver tissue. Saline, NPs-C, NPs, NPs-N, STZ, STZ -SC, STZ - NPs-C, STZ - NPs-C-SC, STZ - NPs, STZ - NPs -SC, STZ - NPs-N, and STZ-NPs-N-SC represent Saline group, NPs-COOH group, NPs group, NPs-NH_2_ group, Fat-STZ-Saline group, Fat-STZ-Saline-SC79 group, Fat-STZ-NP-COOH group, Fat-STZ-NP-COOH-SC79 group, Fat-STZ-NPs group, Fat-STZ-NPs-SC79 group, Fat-STZ-NPs-NH_2_ group and Fat-STZ-NPs-NH_2_-SC79 group, respectively. Results are presented as the mean ± SEM, *n* = 3–8. Statistical significance was analyzed by One-Way ANOVA among multiple groups. * means *p* < 0.05, ** means *p* < 0.01, *** means *p* < 0.001 compared with the saline-vehicle control group; & means *p* < 0.05, && *p* < 0.01 compared with Fat-STZ diabetic group; # means *p* < 0.05, ## means *p* < 0.01, ### means *p* < 0.001 compared with the Fat-STZ-NPs/NH_2_/COOH-SC79; $ means *p* < 0.05, $$ means *p* < 0.01, $$$ means *p* < 0.001 compared between exposure groups; ns means no significant difference

Figure 4A shows that ROS levels in the livers of the T2DM groups were higher than levels in the non-T2DM groups. In the non-T2DM groups, treatment with NPs-NH_2_/COOH resulted in clear increases in ROS concentrations in the liver (*p* < 0.05). In the NPs-NH_2_ group, the ROS concentrations were remarkable higher than those in the NPs group (*p* < 0.05) and NPs-COOH group (*p* < 0.05). In the T2DM groups, treatment with NPs-NH_2_/COOH combined with Fat-STZ induced a marked increase in comparison with the Fat-STZ group (*p* < 0.01); and ROS levels in the Fat-STZ-NPs-NH_2_ group were remarkably higher than in the Fat-STZ-NPs-COOH group (*p* < 0.01). It is worth pointing out that SC79 effectively decreased ROS levels in comparison with the Fat-STZ-NPs-NH_2_ group and with the Fat-STZ-NPs-NH_2_-SC79 group (*p* < 0.05).

As shown in Fig. 4B, the concentrations of MDA in the liver tissues from the T2DM groups were higher than those in the non-T2DM groups. In the non-T2DM groups, the MDA concentrations were not significantly changed. In the T2DM groups, exposure to NPs/NPs-NH_2_/COOH and Fat-STZ induced an obvious rise in MDA levels compared with the Fat-STZ group (*p* < 0.05); but MDA concentrations were insignificant among these exposure groups. Once again, treatment with SC79 was shown to effectively decrease levels of MDA in comparison with the Fat-STZ-NPs-NH_2_ group (*p* < 0.01).

As can be seen in Fig. 4C, GSH levels in the liver tissue of the T2DM groups were lower than those in the non-T2DM groups. In the non-T2DM groups, significant differences did not exist among the exposure groups, nor were there any significant changes among the T2DM groups. Treatment with SC79 effectively increased GSH levels in comparison with the Fat-STZ-NPs/NPs-NH_2_/COOH groups (*p* < 0.05).

### The effect of exposure to PS-NPs modified by different functional groups on histological changes in the liver and pancreas

**Fig. 5:**
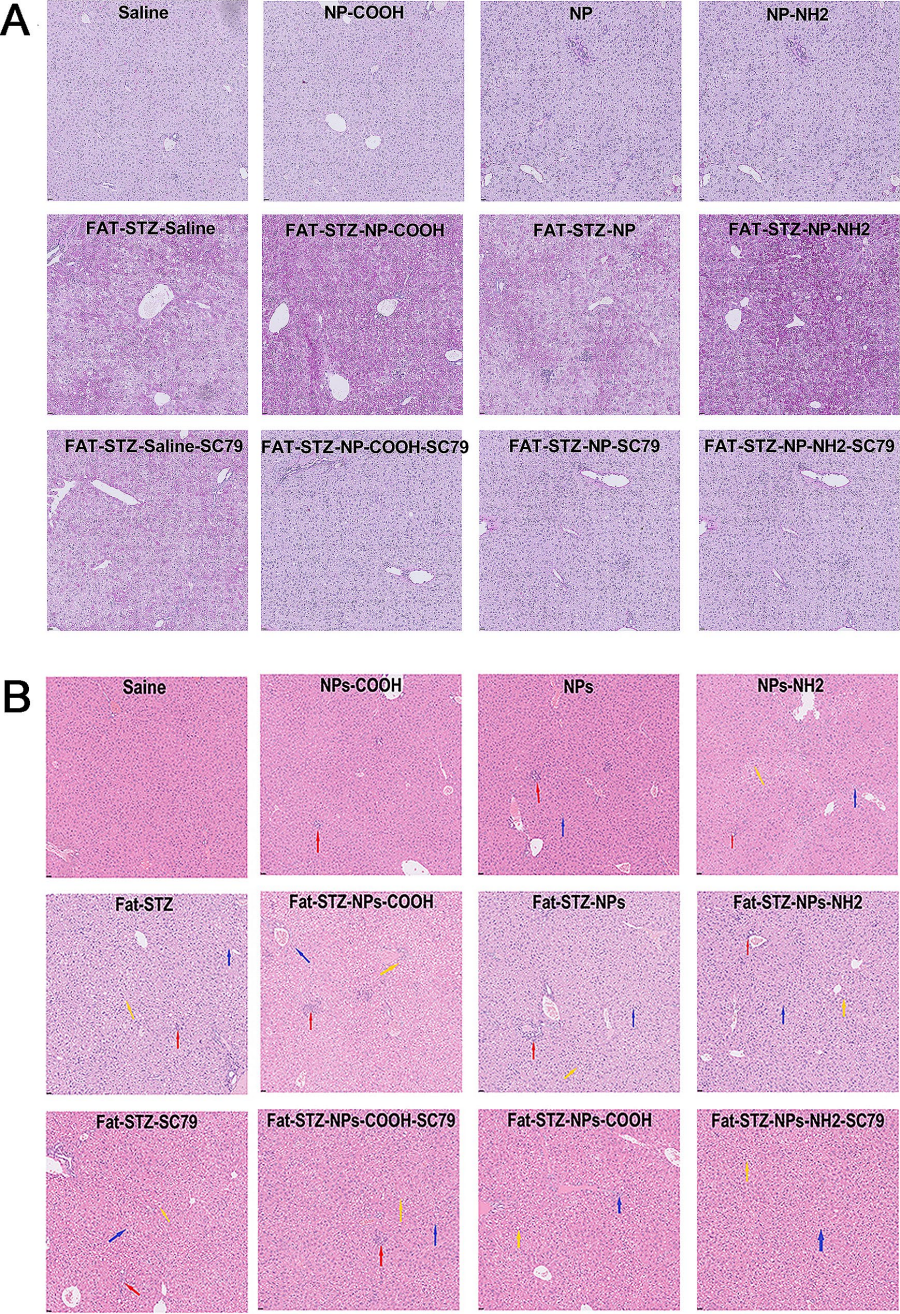
The impact of exposure to PS-NPs with different charges on the liver. (**A**) The periodic acid-schiff (PAS) stain of liver sections. The pictures were magnified 40×; (**B**) H&E staining of liver sections. The red arrow indicates cell necrosis, the yellow arrow indicates lipoatrophy, the blue arrow indicates cellular swelling. The pictures were magnified 40×; *N* = 3, two sections were evaluated per house

Figure 5A presents the results of PAS staining of the liver tissue sections, the depth of color indicates the amount of glycogen. Glycogen accumulation of the T2DM groups was higher than in the non-T2DM groups. Among the non-diabetic groups, the PS-NPs-NH_2_ group had the highest accumulation of glycogen. Among the T2DM groups, PS-NPs/COOH/NH_2_ aggravated glycogen accumulation, with the Fat-STZ-NPs group having the highest glycogen accumulation. Treatment with SC79 attenuated this level of accumulation.

Figure 5B shows that the liver samples from the control group had normal physiological microstructure. For the non-T2DM groups, the liver samples of the PS-NPs/COOH/NH_2_ groups showed a small degree of pathological injury. In the T2DM groups, exposure to PS-NPs/COOH/NH_2_ aggravated histological damage, and of these PS-NPs exposure groups, the PS-NPs-NH_2_ group exhibited the most significant hepatocellular edema and damage. This damage was alleviated in a certain degree after treatment with SC79.

Figure S3 shows that the islets of the pancreas in the control group had well-defined structures, In the PS-NPs/COOH/NH_2_ groups, the islets were irregular in outline and shape, and the structure was blurred and unclear. This damage was especially obvious in the PS-NPs-NH_2_ group. In the diabetic groups, these adverse effects were more obvious. Application of SC79 suppressed these lesions to some extent.

### The effect of exposure to PS-NPs modified by different functional groups on the levels of P-AKTser473 and FoxO1ser256

**Fig. 6:**
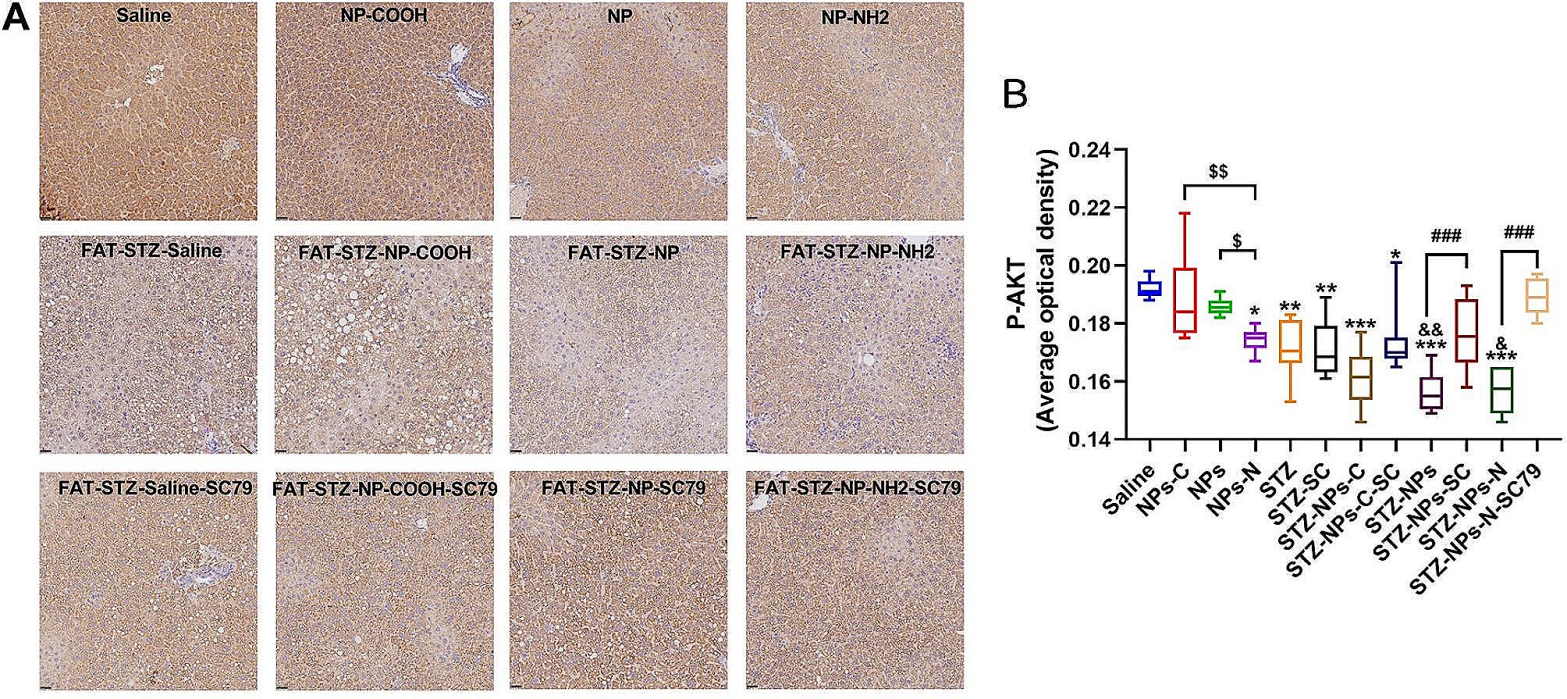
The effect of exposure to PS-NPs with different charges on the levels of P-AKTser473. (A) Immunohistochemistry of P-AKTser473 and the activation scores of P-AKTser473 in the liver. Pictures were magnified 40×; (B) The integrated optical density of each group calculated by Image pro plus 6.0. Saline, NPs-C, NPs, NPs-N, STZ, STZ -SC, STZ - NPs-C, STZ - NPs-C-SC, STZ - NPs, STZ - NPs -SC, STZ - NPs-N, and STZ-NPs-N-SC represent Saline group, NPs-COOH group, NPs group, NPs-NH_2_ group, Fat-STZ-Saline group, Fat-STZ-Saline-SC79 group, Fat-STZ-NP-COOH group, Fat-STZ-NP-COOH-SC79 group, Fat-STZ-NPs group, Fat-STZ-NPs-SC79 group, Fat-STZ-NPs-NH_2_ group and Fat-STZ-NPs-NH_2_-SC79 group, respectively. Results are presented as the mean ± SEM, *n* = 3. Statistical significance was analyzed by One-Way ANOVA among multiple groups. * means *p* < 0.05, ** means *p* < 0.01, *** means *p* < 0.001 compared with the saline-vehicle control group; & means *p* < 0.05, && *p* < 0.01 compared with Fat-STZ diabetic group; # means *p* < 0.05, ## means *p* < 0.01, ### means *p* < 0.001 compared with the Fat-STZ-NPs/NH_2_/COOH-SC79; $ means *p* < 0.05, $$ means *p* < 0.01, $$$ means *p* < 0.001 compared between exposure groups; ns means no significant difference

To investigate the impacts of exposure to PS-NPs/COOH/NH_2_ on the insulin signaling pathways, the levels of P-AKTser473 and P-FoxO1ser256 in liver were visualized by immunohistochemistry. As shown in Fig. 6, the intensity of P-AKTser473 in the non-T2DM groups was greater than seen in the T2DM groups. In the non-T2DM groups, treatment with NPs-NH_2_ significantly degraded the intensity of P-AKTser473 as compared with the saline group (*p* < 0.05); the intensity of P-AKTser473 in the NPs-NH_2_ group was clearly increased when compared with the NPs-COOH/NPs groups (*p* < 0.05). In the T2DM groups, treatment with Fat-STZ-NPs/NPs-NH_2_ significantly decreased the intensity of P-AKTser473 in comparison with the Fat-STZ group (*p* < 0.05); but the differences among these exposure groups were not significant. Treatment with SC79 effectively attenuated levels of P-AKTser473 in comparison with the Fat-STZ-NPs/NH_2_ group (*p* < 0.001).

**Fig. 7:**
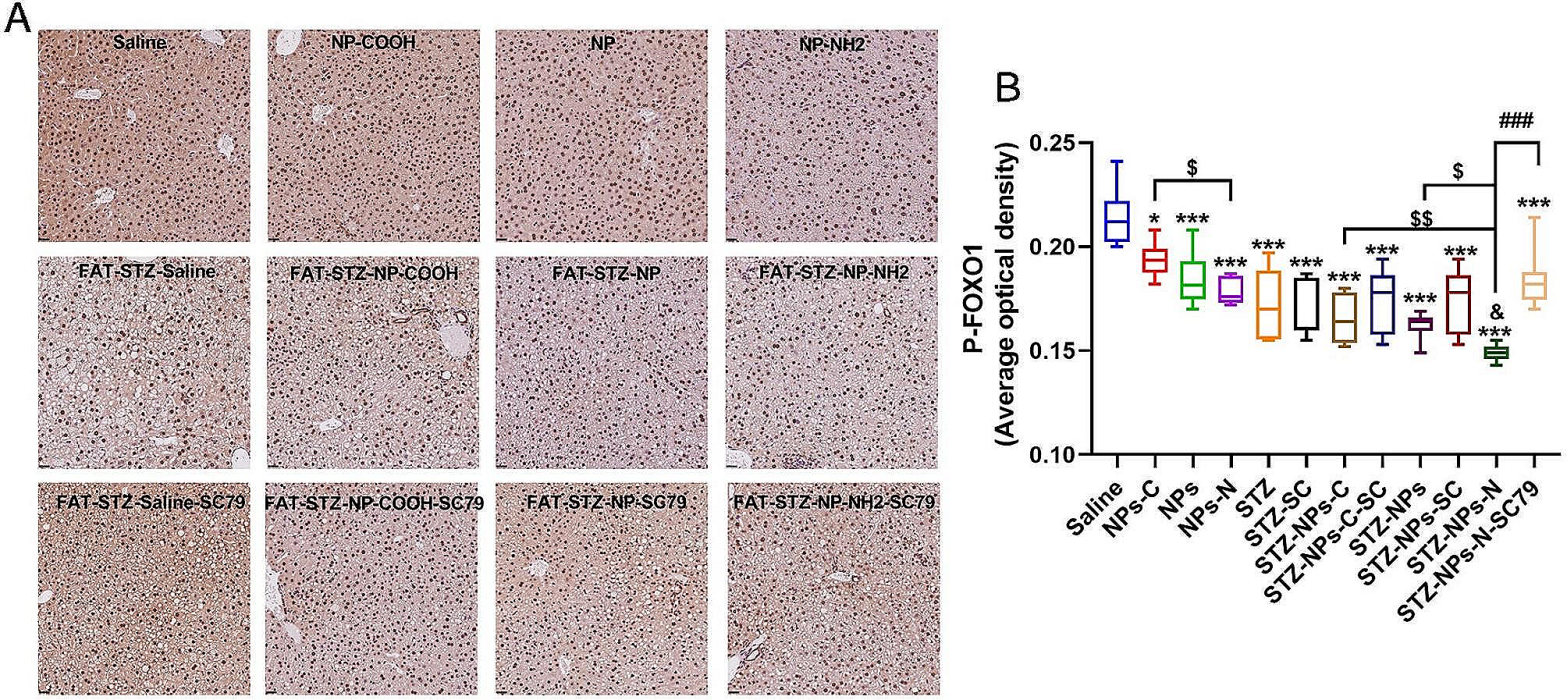
The effect of exposure to PS-NPs with different charges on the levels of FoxO1ser256. (**A**) Immunohistochemistry of P-FoxO1ser256 and the activation scores of P-FoxO1ser256 in the liver. Pictures were magnified 40×. (**B**) The integrated optical density of each group calculated by Image Pro Plus 6.0. Saline, NPs-C, NPs, NPs-N, STZ, STZ -SC, STZ - NPs-C, STZ - NPs-C-SC, STZ - NPs, STZ - NPs -SC, STZ - NPs-N, and STZ-NPs-N-SC represent Saline group, NPs-COOH group, NPs group, NPs-NH_2_ group, Fat-STZ-Saline group, Fat-STZ-Saline-SC79 group, Fat-STZ-NP-COOH group, Fat-STZ-NP-COOH-SC79 group, Fat-STZ-NPs group, Fat-STZ-NPs-SC79 group, Fat-STZ-NPs-NH_2_ group and Fat-STZ-NPs-NH_2_-SC79 group, respectively. Results are presented as the mean ± SEM, *n* = 3. Statistical significance was analyzed by One-Way ANOVA among multiple groups. * means *p* < 0.05, ** means *p* < 0.01, *** means *p* < 0.001 compared with the saline-vehicle control group; & means *p* < 0.05, && *p* < 0.01 compared with Fat-STZ diabetic group; # means *p* < 0.05, ## means *p* < 0.01, ### means *p* < 0.001 compared with the Fat-STZ-NPs/NH_2_/COOH-SC79; $ means *p* < 0.05, $$ means *p* < 0.01, $$$ means *p* < 0.001 compared between exposure groups; ns means no significant difference

The expression of FoxO1ser256 in the non-T2DM groups was higher than that in the T2DM groups (Fig. 7). In the non-T2DM groups, exposure to NPs-COOH/NPs/NPs-NH_2_ significantly reduced the expression of FoxO1ser256 in the liver in comparison with the saline group (*p* < 0.05); and the expression of FoxO1ser256 in the NPs-NH_2_ group was significantly higher in comparison with the NPs-COOH group (*p* < 0.05). Exposure to Fat-STZ-NPs-NH_2_ significantly degraded the expression of FoxO1ser256 in comparison with the Fat-STZ group (*p* < 0.05); and the expression of FoxO1ser256 in the Fat-STZ-NPs-NH_2_ group was higher than that in the Fat-STZ-NPs/NPs-COOH group (*p* < 0.05). SC79 could clearly increase the expression of FoxO1ser256 in comparison with the Fat-STZ-NPs-NH_2_ group (*p* < 0.001).

## Discussion

The biological impact of charged nanomaterials is more unpredictable due to the influence of surface functional groups. For example, positively charged NPs caused mitochondrial damage, oxidative stress and cytotoxicity, to phagocytes [30]. Brake et al. [31] experimented with a number of surface modifications of PS, one of which was a positively charged amino modified PS (PS-NH_2_), which improved electrostatic interactions to serum proteins in comparison to PS [32]. Studies on the impact of NPs with various charges (functional groups) on blood glucose levels, or insulin resistance, have not yielded any significant findings. Which is why, in this work, we investigated the different effects of oral administration of NPs with different charges (functional groups), on FBG, OGTT, ITT, oxidative stress and the insulin pathway in mice. Our findings demonstrated that exposure to PS-NPs with different functional groups (charges) resulted in a marked increase in FBG levels, and exacerbated glucose intolerance and insulin resistance in a mouse model of T2DM. Among these T2DM model groups (PS-NPs/COOH/NH_2_), PS-NPs-NH_2_ exposure caused the most significant increase in FBG, glycogen accumulation, and insulin resistance. Exposure to PS-NPs-NH_2_ alone also result in a notable rise in blood glucose levels, glucose intolerance, and insulin resistance. This suggests that amino modified PS-NPs may lead to more severe T2DM-like lesions than either pristine PS-NPs or carboxyl functionalized PS-NPs. This demonstrated that unmodified NPs may not be as hazardous to organisms as surface amino-modified NPs [13].

Adults may ingest 4 μg–5 g/week of plastic micro-particles through various exposure pathways [33, 34]. A recent study showed that 110,000–370,000 plastic particles were found per liter of bottled water [35], with 90% of these particles being nanoplastics. For this work, mice were exposed to 5 mg/kg/d PS-NPs with different functional groups to compare the impacts of exposure on T2DM.

To investigate the relationship between exposure to PS-NPs with various charges and diabetes, we built a T2DM model using a high-fat diet and STZ injection. Increased water intake, increased urination, and weight loss are typical symptoms associated with diabetes mellitus [36]. After exposure for 9 weeks, we found that the diabetic model groups lost more weight than the non-model groups. We also observed an increase in water intake and urination in the diabetic model group of mice by comparing with the saline control group and the non-diabetic groups.

T2DM, is characterized by a progressive loss of insulin sensitivity, commonly referred to as insulin resistance. This condition is associated with elevated levels of insulin, leading to compensatory hyperinsulinemia and fasting hyperglycemia [37].. Our findings suggested that the T2DM mice had higher FBG levels than the control group. Also, levels of HOMA-IR, OGTT, ITT in the diabetic groups were higher than in the non-diabetic groups. This indicated that a high fat diet combined with STZ injection were successful in building a T2DM model.

PS-NPs-NH_2_ activates the apoptotic pathway through lysosomal disruption and ROS production, stimulating cathepsins and caspases [38]. It has long been assumed that oxidative stress is a primary factor in the development of insulin resistance, progressive-cell dysfunction, poor glucose utilization, and, ultimately, T2DM. Chronic hyperglycemia increases ROS production, and lipid peroxidation in tissues, both of which limit insulin function [39]. So, we looked at ROS levels in the liver as well as at other oxidative indicators. We found that exposure to PS-NPs/NH_2_/COOH does affect the balance of the hepatic redox system.

The impact of oxidative damage is critical for hepatic insulin transmission [40]. A rise in ROS levels inhibits the recruitment and activation of insulin receptors, IRS-1 and IRS-2, by reducing the binding of ligands to these receptors. Additionally, it impairs the downstream PI3K-protein kinase B (AKT) signaling pathway, which is essential for glucose metabolism [41]. The PI3K/AKT signaling pathway can also activate downstream Nrf2 signaling, thereby exerting an antioxidant stress effect [42]. The liver is a major site of glucose metabolism and is also the key target organ for IR treatment [43]. The liver plays a crucial role in regulating the balance of systemic metabolism. Glucose metabolism in the liver is balanced by insulin and also includes lipid metabolism [44]. When blood glucose levels increase, insulin from the pancreas binds to hepatic insulin receptors, causing them to undergo autophosphorylation. Following activation of the PI3K-Akt signaling pathway by IRS recruitment and phosphorylation, the liver is involved in regulating several metabolic pathways, including hepatic gluconeogenesis, glycogen synthesis, lipid synthesis, and glycolysis [45].. Studies have suggested that the IRS/PI3K-Akt/FoxO1 signaling pathway plays a crucial role in regulating hepatic glucose metabolism by insulin. Forkhead box protein O1 (FoxO1) is phosphorylated to enhance nuclear exclusion, which in turn suppresses the production of important rate-limiting gluconeogenesis enzymes such as PEPCK, G6Pase, and FBP1, eventually suppressing HGP [45]. We therefore determined the levels of P-AKTser473 and P-FoxO1ser256 in the liver, and found that the phosphorylated levels of AKTser473/ FoxO1ser256 were higher in the non-modeling group than in the diabetic modeling group. Notably, exposure to NPs-NH_2_ was found to significantly decrease phosphorylation levels of AKTser473 and FoxO1ser256 in the liver.

To verify the involvement of the PI3K-Akt signaling pathway on T2DM like lesions induced by exposure to PS-NPs with different functional groups (charges), we used SC79 to activate AKT. The use of SC79 did indeed effectively increase the level of P-AKTser473 and P-FoxO1ser256, and after administration of SC79, we found a noticeable increase in the levels of insulin, and a decline in the levels of HOMA-IR. This effectively alleviated the FBG level elevation and glycogen accumulation, and attenuated the oxidative stress and damage to the liver and pancreas induced by PS-NPs with different functional groups or charges. These results also confirmed our previous finding, that the PI3K-Akt signaling pathway may be involved in the appearance of T2DM-like lesions after PS-NPs exposure [27]. We can draw the conclusion from this work, that exposure to PS-NPs with different charges can harm the liver and pancreas, result in oxidative stress, affect how AKT and GSK3 are phosphorylated, and alter blood glucose levels and other markers. Of the three PS-NPs we examined, PS-NPs-NH_2_ appeared to have the highest toxicity.

## Conclusions

To the best of our current understanding, this is the first exploration into the effects and mechanisms of PS-NPs with different functional groups or charges, on T2DM-like lesions in mice. Among the T2DM model groups (PS-NPs/COOH/NH_2_), the PS-NPs-NH_2_ group was found to have the most significant impact on the elevation of FBG levels, glycogen accumulation, and insulin resistance. This suggests that amino-modified PS-NPs may cause more serious T2DM-like lesions than pristine PS-NPs or carboxyl-modified PS-NPs.

Exposure to PS-NPs with different charges can impair the phosphorylation of AKT and FoxO1, and treatment with SC79 effectively rescued this process, and alleviated T2DM-like lesions. This demonstrated that the underlying mechanisms of T2DM-like lesions induced by exposure to PS-NPs with different charges involved the inhibition of P-AKT/P-FoxO1. This study has not only revealed the effects and mechanisms of NPs with different functional groups or charges on T2DM, but also provides a new perspective for correctly evaluating the health risks of NPs.

### Electronic supplementary material

Below is the link to the electronic supplementary material.

Supplementary Material 1

## Data Availability

All relevant data are included in the manuscript and supporting information files.
