# Supplementary material for: Polystyrene nanoplastics with different functional groups and charges have different impacts on type 2 diabetes
Source: Part Fibre Toxicol. 2024 Apr 24;21:21. doi: 10.1186/s12989-024-00582-w (PMC11044502; doi:10.1186/s12989-024-00582-w)
Supplement: Supplementary file 1 — Supplementary Material 1 [file 12989_2024_582_MOESM1_ESM.docx]

**Supplementary Material**

**Polystyrene nanoplastics with different functional groups and charges have different impacts on type 2 diabetes**

Yunyi Wang, Ke Xu, Xiao Gao, Zhaolan Wei, Qi Han, Shuxin Wang, Wanting Du, Mingqing Chen*

Hubei Key Laboratory of Genetic Regulation and Integrative Biology, School of Life Sciences, Central China Normal University, Wuhan 430079, Hubei, China.

*Corresponding Author at: School of Life Sciences, Central China Normal University, Wuhan 430079, China.

E-mail address: [chenmq@mail.ccnu.edu.cn](mailto:chenmq@mail.ccnu.edu.cn)

**Figure S1:**


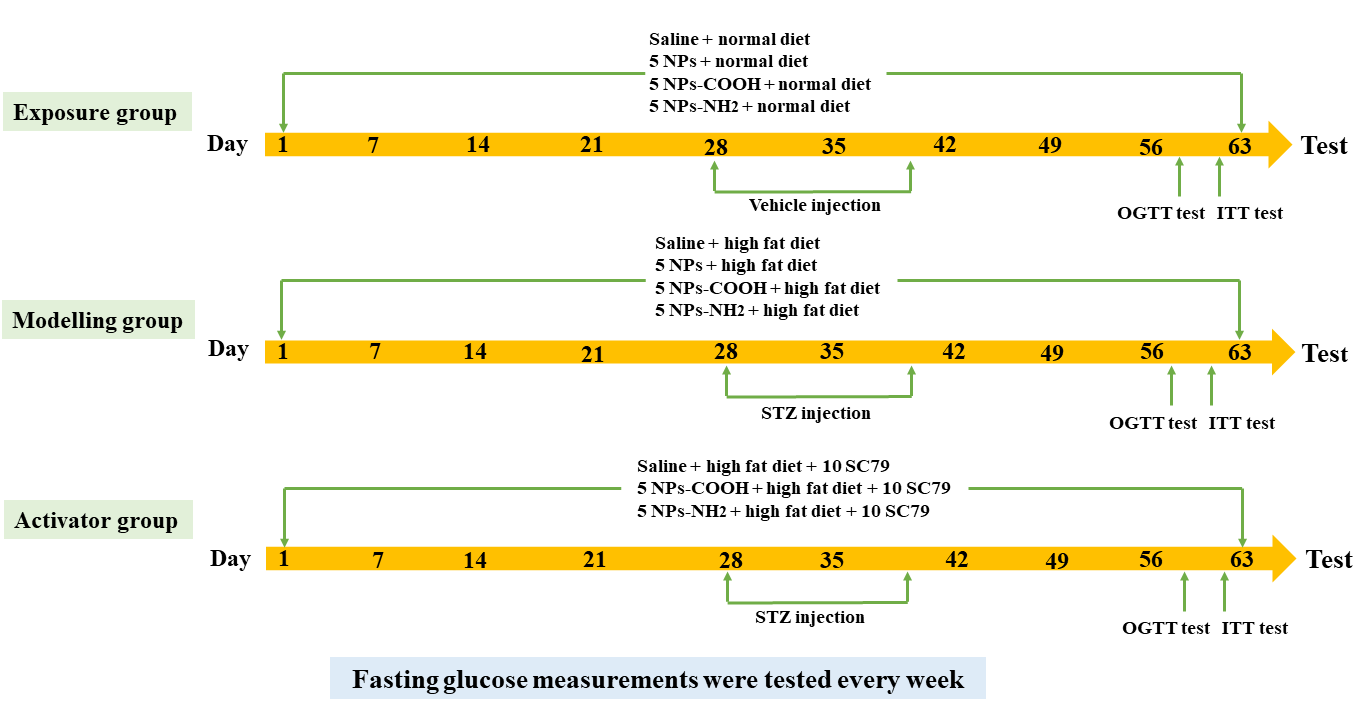


**Fig. S1. Experimental design.** Group (1) saline group; (2) 5 mg/kg/day PS-NP-COOH; (3) 5 mg/kg/day PS-NP; (4) 5 mg/kg/day PS-NP-NH_2_; (5) saline + high fat diet; (6) 5 mg/kg/day PS-NP-COOH + high fat diet; (7) 5 mg/kg/day PS-NPs + high fat diet; (8) 5 mg/kg/day PS-NP-NH_2_ + high fat diet; (9) saline + high fat diet + SC79; (10) 5 mg/kg/day PS-NP-COOH + high fat diet + SC79; (11) 5 mg/kg/day PS-NPs + high fat diet + SC79; (12) 5 mg/kg/day PS-NP-NH_2_ + high fat diet + SC79. Intraperitoneal injection of STZ: every two days. Intraperitoneal injection of SC79: every three days. N=10 (each group). STZ: streptozocin; NPs: nanoplastics; SC79: HY-18749, a selective AKT activator; OGTT: oral glucose tolerance test; ITT: insulin tolerance test.

**Fig. S2:**


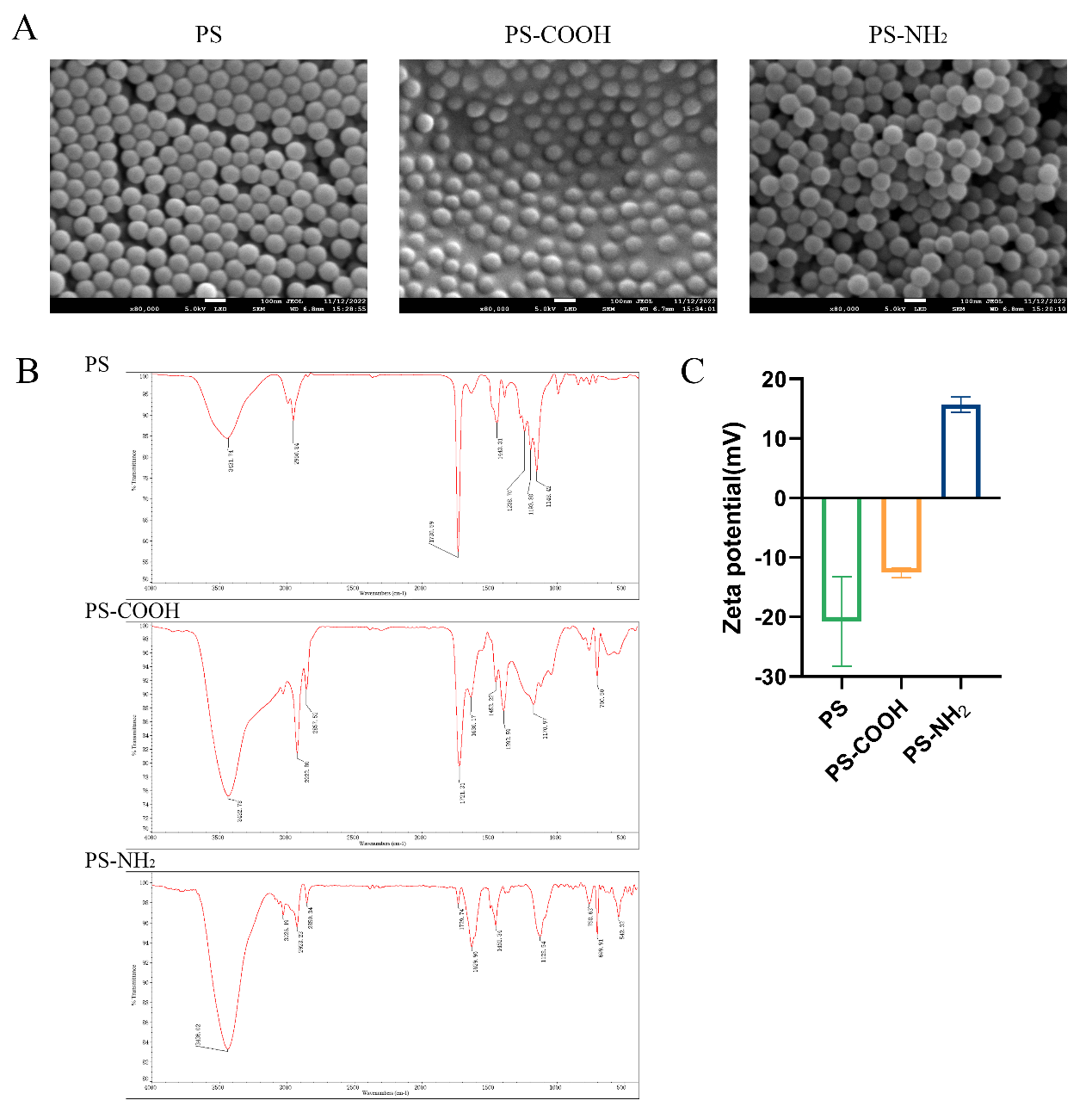


**Fig. S2.** **Characteristics of PS-NPs with different functional groups.** A. SEM images; B. Infrared spectrogram; C. Zeta potentials of three PS-NPs in saline

**Figure S3:**


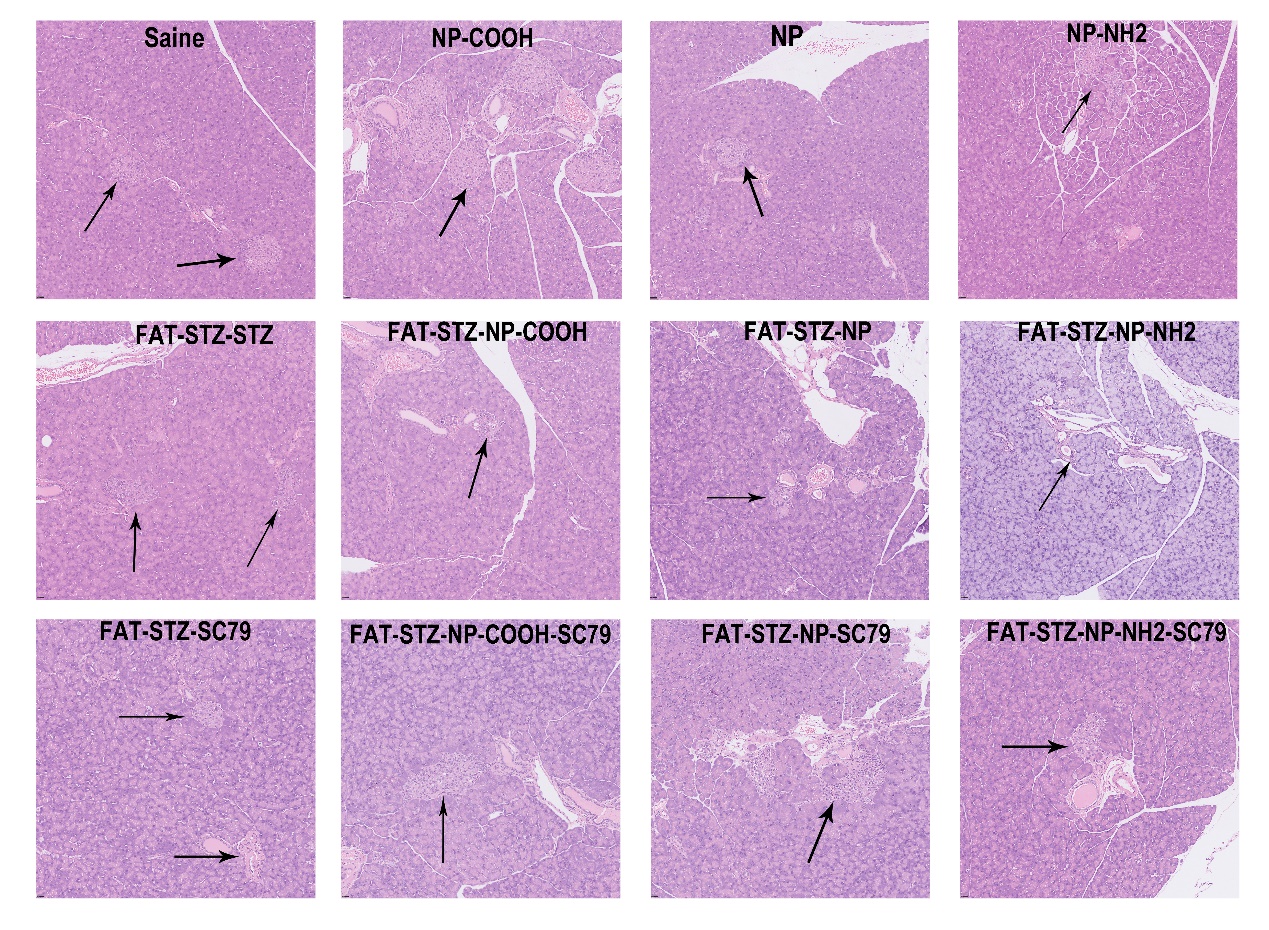


**Fig. S3.**  **The impact of exposure to PS-NPs with different charges on the pancreas**. The black arrow indicates the pancreas islets. The pictures are magnified 40×. N=3, two sections were evaluated per mouse. PAS: periodic acid-schiff stain.
